# Predicting Clinical Outcome in Expanded Criteria Donor Kidney Transplantation: A Retrospective Cohort Study

**DOI:** 10.1177/2054358120924305

**Published:** 2020-06-24

**Authors:** Paramita Saha-Chaudhuri, Carly Rabin, Jean Tchervenkov, Dana Baran, Justin Morein, Ruth Sapir-Pichhadze

**Affiliations:** 1 Department of Epidemiology, Biostatistics & Occupational Health, McGill University, Montréal, QC, Canada; 2 Department of Pediatrics, State University of New York Downstate Medical Center, Brooklyn, NY, USA; 3 Department of Surgery, McGill University, Montréal, QC, Canada; 4 Division of Nephrology and the Multi Organ Transplant Program, Royal Victoria Hospital, McGill University Health Centre, Montréal, QC, Canada; 5 Department of Medicine, University of Western Ontario, London, ON, Canada; 6 Centre for Outcomes Research & Evaluation, Research Institute of the McGill University Health Centre, Montréal, QC, Canada

**Keywords:** kidney transplantation, survival, extended criteria donors, histopathology, Kidney Donor Risk Index, estimated glomerular filtration rate

## Abstract

**Background:**

The gaps in organ supply and demand necessitate the use of expanded criteria donor (ECD) kidneys.

**Objective:**

To identify which pre-transplant and post-transplant predictors are most informative regarding short- and long-term ECD transplant outcomes.

**Design:**

Retrospective cohort study.

**Setting:**

Single center, Quebec, Canada.

**Patients:**

The patients were 163 consecutive first-time ECD kidney only transplant recipients who underwent transplantation at McGill University Health Centre (MUHC) between January 1, 2008 and December 31, 2014 and had frozen section wedge procurement biopsies.

**Measurements:**

Short-term graft outcomes, including delayed graft function and 1-year estimated glomerular filtration rate (eGFR), as well as long-term outcomes including all-cause graft loss (defined as return to dialysis, retransplantation, and death with function).

**Methods:**

Pre-transplant donor, recipient, and transplant characteristics were assessed as predictors of transplant outcomes. The added value of post-transplant predictors, including longitudinal eGFR, was also assessed using time-varying Cox proportional hazards models.

**Results:**

In univariate analyses, among the pre-transplant donor characteristics, histopathologic variables did not show evidence of association with delayed graft function, 1-year post-transplant eGFR or all cause graft loss. Recipient age was associated with all-cause graft loss (hazard ratio: 1.038 [95% confidence interval: 1.002-1.075] and the model produced only modest discrimination (C-index: 0.590; standard error [SE]: 0.045). Inclusion of time-dependent post-transplant eGFR improved the model’s prediction accuracy (C-index: 0.711; SE = 0.047). Pre-transplant ECD characteristics were not associated with long-term survival, whereas post-transplant characteristics allowed better model discrimination.

**Limitations:**

Single-center study, small sample size, and potential incomplete capture of all covariate data.

**Conclusions:**

Incorporation of dynamic prediction models into electronic health records may enable timely mitigation of ECD graft failure risk and/or facilitate planning for renal replacement therapies. Histopathologic findings on preimplantation biopsies have a limited role in predicting long-term ECD outcomes.

**Trial registration:**

Not applicable.

## Abrégé

**Contexte:**

Les écarts entre l’offre et la demande d’organes nécessitent le recours à des donneurs répondant à des critères élargis (DCÉ).

**Objectif:**

Déterminer les prédicteurs pré- et post-transplantation qui s’avèrent les plus instructifs quant aux résultats à court et à long terme des greffes d’organes provenant de DCÉ.

**Type d’étude:**

Étude de cohorte rétrospective

**Cadre:**

Étude monocentrique tenue au Québec (Canada).

**Sujets:**

L’étude porte sur 163 patients consécutifs greffés d’un rein seulement au center universitaire de santé McGill (CUSM) entre le 1^er^ janvier 2008 et le 31 décembre 2014. L’organe reçu provenait d’un DCÉ et la biopsie avait été effectuée sur des sections congelées de l’organe.

**Mesures:**

Les résultats à court terme, notamment la reprise retardée de la fonction du greffon et le débit de filtration glomérulaire estimé (DFGe) après un an. Les résultats à long terme dont la perte du greffon toute cause (retour en dialyse, retransplantation ou décès avec greffon fonctionnel).

**Méthodologie:**

Les caractéristiques du donneur, du receveur et de la transplantation ont été examinées avant l’intervention comme prédicteurs de l’issue de la greffe. Des modèles des risques proportionnels de Cox variant dans le temps ont servi à évaluer la valeur ajoutée des prédicteurs post-greffe, notamment du DFGe longitudinal.

**Résultats:**

Dans l’analyse univariée des caractéristiques pré-transplantation, les variables histopathologiques n’ont montré aucune association avec la fonction retardée du greffon, le DFGe un an après la greffe ou la perte du greffon. L’âge du receveur a été associé à la perte du greffon toute cause (RR : 1,038 [IC 95 % : 1,002-1,075]) et le pouvoir discriminant du modèle s’est avéré modeste (indice C : 0,590; ÉT = 0,045). L’inclusion du DFGe post-greffe en fonction du temps a amélioré la précision prédictive du modèle (indice C : 0,711; ÉT = 0,047). Les caractéristiques pré-greffe du DCÉ n’ont pas été associées à la survie à long terme, alors que les caractéristiques post-greffe ont permis d’améliorer le pouvoir discriminant du modèle.

**Limites:**

Étude monocentrique sur un faible échantillon et dont la saisie des données sur les covariables est potentiellement incomplète.

**Conclusion:**

L’incorporation de modèles prédictifs dynamiques aux dossiers médicaux électroniques pourrait permettre, en temps opportun, d’atténuer les risques de défaillance du greffon provenant d’un DCÉ et faciliter la planification d’une thérapie de remplacement rénal. Les résultats histopathologiques des biopsies pré-transplantation jouent un rôle mineur pour prédire les résultats à long terme des DCÉ.

**Enregistrement de l’essai:**

Sans objet

## What was known before

To bridge the gap between organ supply and demand, the practice of accepting expanded criteria donor (ECD) kidneys for transplantation has become more prevalent. Clinical donor characteristics and histopathology are used to inform on allograft quality.

## What this adds

Long-term ECD transplant outcomes are not informed by donor characteristics alone; rather, an interplay with recipient characteristics predicts ECD transplant outcomes. Of the pre-transplant characteristics, recipient age was most predictive of long-term transplant outcomes with longitudinal post-transplant eGFR improving model performance.

## Introduction

Over the years, there has been a universal shortage and an ever-increasing need for donor kidneys. Donor and recipient age have also been steadily rising.^1-4^ To bridge the gap between organ supply and demand, the practice of accepting expanded criteria donor (ECD) kidneys for transplantation has become more prevalent. In this context, it is crucial to understand the determinants of outcomes in kidney transplant recipients (KTR) who receive ECD transplants.^5^

Various donor characteristics measured at the time of organ transplantation have been linked to long-term kidney transplant outcomes. The Kidney Donor Risk Index (KDRI), for example, estimates the relative risk of post-transplant kidney graft failure (in an average, adult recipient) associated with each donor.^6^ The KDRI considers donor age, height, weight, ethnicity, history of hypertension or diabetes, cause of death, serum creatinine, history of hepatitis C, and donation after cardio-circulatory death (DCD).^7^

In addition to clinical characteristics, frozen section wedge procurement biopsies (FSWB) have been used to guide decisions on organ utilization.^8^ In a recent systematic review, Wang et al^9^ reported on findings from 47 manuscripts published between 1994 and 2014. Quality assessment established that many studies were prone to selection bias because decisions on utilization or discard of ECD kidneys often relied on the perceived quality of procurement biopsies. Studies were also prone to bias related to inconsistent conduct of biopsies and incomplete account for confounders. Some studies reported on paraffin-embedded implantation biopsies rather than FSWB and some studies did not exclusively evaluate ECD transplants but also included biopsies from living donors or very young deceased donors.

In the absence of a clear consensus on what should determine the utilization of ECD kidneys,^10^ our objective was to identify which donor (eg, KDRI, FSWB findings, and terminal estimated glomerular filtration rate [eGFR]), transplant, and recipient characteristics were independent predictors of the short-term (ie, delayed graft function [DGF] and 1-year eGFR) and long-term outcomes (graft failure and death). Although current standards for long-term monitoring rely on eGFR, the role of dynamic eGFR monitoring post-transplant in determining transplant outcomes has not been defined to date. For this reason, in addition to baseline donor, recipient, and transplant characteristics, we also assessed the incremental value of longitudinal eGFR measurements in predicting long-term transplant outcomes.

## Results

### Study Population

**Figure 1.**
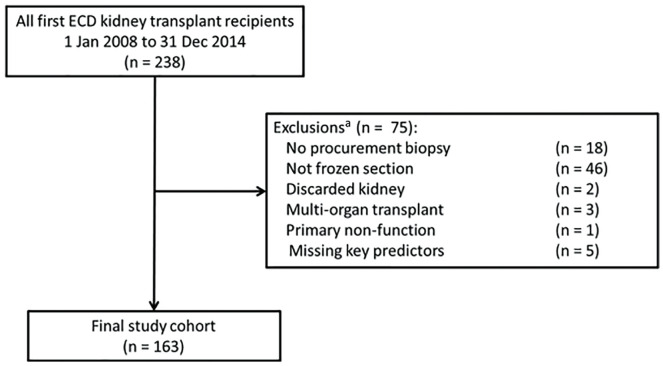
Study flow diagram. *Note.* ECD = expanded criteria donor. ^a^Participants may have been ineligible for participation for more than 1 reason (eg, 3 participants experienced primary nonfunction).

A total of 238 patients underwent negative cross-match kidney transplants at McGill University Health Centre (MUHC) between 2008 and 2014. After application of the exclusion criteria, 163 unique recipients and 135 unique donors (kidneys from 28 donors were transplanted into 2 recipients) were included in the analytic cohort (Figure 1). Recipient, donor, and transplant characteristics of the analytic cohort are presented in Table 1.

**Table 1.** Baseline Recipient, Transplant, and Donor Characteristics.

Unit		Mean (SD) or No. (%)^a^
Recipient characteristics (n = 163)		
Age, years		62.57 (9.49)
Height, cm		167.65 (10.06)^b^
Weight, kg		78.20 (15.42)^b^
Sex, No. (%)		
Female		53 (33)
Male		110 (67)
Race, No. (%)		
White		117 (72)
Other^c^		45 (28)
Time on dialysis, days		1371 (776)
Cause of end-stage renal disease, No. (%)		
Diabetic nephropathy		51 (31)
Cystic kidney disease		25 (15)
Other		87 (53)
Transplant characteristics (n = 163)		
Transplant era, No. (%)		
2008-2011		75 (46)
2012-2014		88 (54)
Cold ischemia time, hours		18.74 (7.00)
Pulsatile machine perfusion, No. (%)		
Yes		146 (90)
No		15 (9)
Missing		2 (1)
Induction therapy, No. (%)		
Non-lymphocyte-depleting^d^		16 (10)
Lymphocyte-depleting^e^		145 (89)
None		2 (1)
Maintenance immunosuppression, No. (%)		
Triple agent (tacrolimus, mycophenolate, prednisone)		100 (61)
Dual agent (tacrolimus, mycophenolate)		59 (36)
Other		4 (2)
Human leukocyte antigen mismatch, No. (%)		
1-2		16 (10)
3-4		89 (55)
5-6		58 (36)
Donor characteristics (n = 135)		
Age, y		65.28 (7.01)
Height, cm		166.51 (14.75)
Weight, kg		78.59 (15.61)
Sex, No. (%)		
Female		59 (44)
Male		74 (55)
Missing		2 (1)
Race, No. (%)		
White		130 (96)
Other		2 (1)
Missing		3 (3)
Hypertension, No. (%)		75 (56)
Diabetes, No. (%)		26 (19)
Hepatitis C infection, No. (%)		0 (0)
Donation after cardio-circulatory death (DCD), No. (%)		8 (6)
History of smoking, No. (%)		98 (73)
Terminal creatinine, mmol/L		66 (27)
Terminal estimated glomerular filtration by Chronic Kidney Disease Epidemiology Collaboration		92 (18)^f^
Kidney Donor Risk Index (KDRI)		1.81 (0.39)
Histology characteristics		
Glomerular global sclerosis, No. (%)	0	22 (16)
	1	99 (73)
	2	12 (9)
	3	2 (1)
Arteriolar hyalinosis, No. (%)	0	113 (84)
	1	17 (13)
	2	2 (1)
	3	3 (2)
Interstitial fibrosis, No. (%)	0	91 (67)
	1	34 (25)
	2	9 (7)
	3	1 (1)
Tubular atrophy, No. (%)	0	96 (71)
	1	35 (26)
	2	4 (3)
	3	0 (0)
Arteriolosclerosis, No. (%)	0	94 (70)
	1	29 (21)
	2	10 (7)
	3	2 (1)
Remuzzi, No. (%)	Mild (0-3)	105 (78)
	Moderate (4-6)	28 (21)
	Severe (7-12)	2 (1)
Karpinski, No. (%)	Mild (0-3)	18 (13)
	Moderate (4-6)	107 (79)
	Severe (7-12)	10 (7)

a Because of rounding, the sum of proportion for a categorical covariate may not be equal to 100.

b Missing for 4 recipients.

c Self-reported race was missing in 1 recipient.

d Non-lymphocyte-depleting induction agent included basiliximab.

e Lymphocyte-depleting induction agents were antithymocyte globulin (prior to 2011) and alemtuzumab (subsequent to 2011).

f Missing for 5 donors because of missing sex (N = 2) and missing race (N = 3).

Among the transplants, 46% were conducted on or before 2011. The recipients were primarily white (72%), males (67%) with an average age of 62 years (range: 27-80 years). The cause of end-stage renal disease (ESRD) was diabetic nephropathy (31%), cystic kidney disease (15%), or other (53%), and the average time on dialysis was 43 months.

The donors were primarily white (96%), males (55%) with an average age of 65 years (range: 50-85 years). Hypertension and diabetes were present in 56% and 19% of donors, respectively. Only 6% were DCD. Seventy-three percent of donors had a history of smoking. Average terminal donor creatinine was 66 mmol/L (range: 32-190) corresponding to an eGFR of 91 mL/min (range: 32.32-127.13). The mean KDRI for donors was 1.81 (range: 1.22-3.49).

Most grafts (90%) underwent pulsatile machine perfusion. Mean cold ischemia time was 18.74 hours. Eighty-nine percent of KTR received lymphocyte-depleting induction agents and 61% were on a triple-agent maintenance immunosuppression regimen.

### Short-Term Outcome Models

#### DGF

Forty recipients experienced DGF. Recipient age and sex were included in the model a priori. Continuous variables (donor age, height, weight, terminal eGFR, and KDRI) and categorical variables (donor sex, race, hypertension, diabetes, DCD status, smoking, and biopsy findings [glomerular global sclerosis (GS), arteriolar hyalinosis (AH), interstitial fibrosis (CI), tubular atrophy (CT), arteriolosclerosis (CV), Remuzzi and Karpinski scores]) were considered for inclusion in the model. To avoid model overfitting,^11^ we chose to include at most 3 characteristics, and these were chosen by univariate screening with a *P* value threshold of .2, resulting in a multivariate logistic regression model for DGF including recipient age and sex as well as donor age, sex, and DCD status. The odds ratios (ORs) and corresponding 95% confidence intervals (CIs) are shown in Table 2. Only donor DCD status was statistically significant with an OR of 4.544 (95% CI: 1.074-20.611). Recipients of male donors were at higher odds of DGF as compared with recipients of female donors, but the association was not statistically significant. There was a trend toward higher odds of DGF the higher the recipient and donor age, but this did not meet the assigned threshold for statistical significance. Of the original cohort, only 3 recipients experienced primary non-function (PNF). Therefore, we could not fit a prediction model for this outcome and KTR who experienced PNF were excluded from the study.

**Table 2.** Odds Ratios for Delayed Graft Function by Pre-transplant Donor, Recipient, and Transplant Characteristics.

Characteristics	Univariate screening analysis	Multivariate analysis	
	*P* value	Odds ratio	95% CI
Recipient age	^a^	1.030	0.983-1.084
Recipient sex (male)	^a^	1.086	0.477-2.569
Donor age	0.102	1.045	0.986-1.110
Donor sex (male)	0.107	1.915	0.875-4.362
Donation after cardio-circulatory death	0.0381	4.544	1.074-20.611
C-index	NA	0.687 (SE = 0.048)	

*Note.* CI = confidence interval.

a Included in multivariate model a priori.

#### One-year post-transplant eGFR

When modeling 1-year post-transplant eGFR, we included subjects who survived past 1-year post-transplant (N = 145 recipients). The eGFR data are presented in Supplementary Information 1. In addition to recipient age and sex, we considered the same donor characteristics as for the DGF model. The multivariate linear regression model included donor characteristics that showed association with the eGFR (*P* value threshold of .2) in the univariate selection. These characteristics included donor age, height, KDRI, sex, history of diabetes, DCD, history of smoking, histological lesions (GS and CT), and histological summary scores (Remuzzi and Karpinski). Parameter estimates, standard errors (SEs), and 95% CIs from the multivariate model are presented in Table 3. Of these characteristics, donor history of diabetes and DCD status were deemed statistically significant predictors.

**Table 3.** Results of Multivariate and LASSO Linear Regression Models for 1-Year Estimated Glomerular Filtration Rate.

Variable	Multivariate linear regression			LASSO (lambda= 3.42)
	Estimate	SE	95% confidence interval	Estimate
Intercept	55.504	25.160	6.192 to 104.816	54.883
Recipient age	0.134	0.173	−0.205 to 0.472	*
Recipient sex = female (ref)	—	—	—	—
Recipient sex = male	−0.846	3.505	−7.716 to 6.023	*
Donor age	−0.817	0.527	−1.850 to 0.215	*
Donor height	0.225	0.162	−0.092 to 0.542	*
Kidney Donor Risk Index	5.514	20.831	−35.314 to 46.341	−16.231
Donor sex = female (ref)	—	—	—	—
Donor sex = male	1.319	3.666	−5.867 to 8.504	*
Donor DM = no (ref)	—	—	—	—
Donor DM = yes	−12.341	5.314	−22.757 to −1.926	*
Donor DCD = no (ref)	—	—	—	—
Donor DCD = yes	−17.064	7.509	−31.782 to −2.346	*
Donor smoking = no (ref)	—	—	—	—
Donor smoking = yes	5.169	3.724	−2.130 to 12.469	*
GS = 0 (ref)	—	—	—	—
GS = 1	−19.048	12.060	−42.686 to 4.590	*
GS = 2	−20.200	13.490	−46.640 to 6.240	*
GS = 3	−55.810	29.061	−112.768 to 1.149	*
CT = 0 (ref)	—	—	—	—
CT = 1	−1.890	5.305	−12.287 to 8.507	*
CT = 2	−19.453	13.771	−46.443 to 7.537	*
CT = 3^a^	—	—	—	—
Remuzzi = 0-3 (ref)	—	—	—	—
Remuzzi = 4-6	−3.860	5.702	−15.036 to 7.317	*
Remuzzi = 7-12	−4.392	21.926	−47.366 to 38.582	*
Karpinski = 0-3 (ref)	—	—	—	—
Karpinski = 4-6	14.135	13.118	−11.575 to 39.845	*
Karpinski = 7-12	29.091	17.563	−5.333 to 63.515	*

*Note.* Estimates for variables marked with (*) in LASSO are zero. LASSO = least absolute shrinkage and selection operator; DM = diabetes mellitus; DCD = donation after cardio-circulatory death; GS = glomerular global sclerosis; CT = tubular atrophy.

a Only 1 subject had a CT and GS score of 3, so no coefficient for CT was returned.

We also fit a least absolute shrinkage and selection operator (LASSO) linear regression model^12^ as a sensitivity analysis. The LASSO model found only KDRI to be an important predictor of 1-year eGFR. The results are shown in Table 4.

**Table 4.** Relative Hazard of All-Cause Graft Loss by Pre-transplant Donor, Recipient, and Transplant Characteristics.

Characteristics	Hazard ratio	95% confidence interval
Recipient age	1.038	1.002-1.075
Recipient sex (male)	0.732	0.401-1.336
Cold ischemia time	1.016	0.975-1.058
Recipient’s cause of end-stage renal disease (diabetic nephropathy)	1.598	0.892-2.864
Donor terminal estimated glomerular filtration rate	1.008	0.991-1.024
C-index	0.590 (SE = 0.045)	

### Long-Term Outcome Models

**Figure 2.**
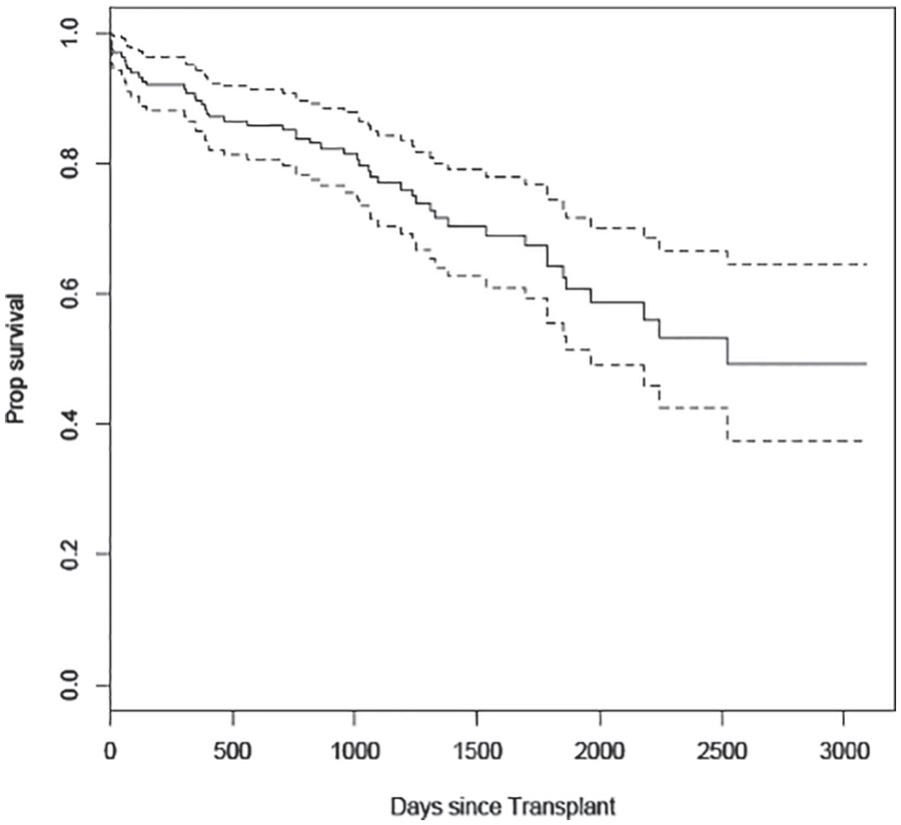
Kaplan-Meier survival curve (solid line) and 95% confidence interval (dashed line) for all-cause graft loss. *Note.* Prop. = proportion.

Over the duration of the study, 50 KTR experienced all-cause graft loss events (27 graft failures and 23 deaths with a functioning graft). The median graft survival time was 6.91 years. The Kaplan-Meier survival curve for all-cause graft failure is presented in Figure 2.

#### Pre-transplant graft survival model

When assessing the association between pre-transplant recipient, donor, and transplant characteristics with all-cause graft loss using univariate Cox proportional hazards models, recipient age (hazard ratio [HR]: 1.03; 95% CI: 1.00-1.07) and diabetic nephropathy as the cause of ESRD (HR: 1.7; 95% CI: 0.96-3.00) were the pre-transplant variables that demonstrated a strong association with all-cause graft loss. Histologic lesions and KDRI (HR: 1.80; 95% CI: 0.41-7.84) were not associated with this outcome. In addition, the scaled Schoenfeld residual analysis showed strong nonproportionality of hazard, but no clear biologically plausible association pattern. Therefore, the final multivariate Cox proportional hazards model included recipient age, sex, and diabetic nephropathy as causes of ESRD along with donor pre-procurement eGFR and cold ischemia time (Table 4).

When assessing the association between pre-transplant characteristics with death-censored graft failure (DCGF), none of the characteristics (including histopathology and KDRI) were statistically significantly associated with DCGF. Following our modeling strategies for all-cause graft loss, we built a pre-transplant model with recipient age and sex as covariates. The model demonstrated limited discrimination (C-index = 0.535; SE = 0.062).

#### Post-transplant graft survival model

To account for post-transplant predictors of long-term kidney transplant outcomes, we also assessed the incremental value of DGF and longitudinal post-transplant eGFR estimates over and above pre-transplant characteristics by fitting a multivariate Cox proportional hazards model. The HRs and 95% CIs are presented in Table 5. While donor terminal eGFR, donor sex, cold ischemia time, and recipients’ cause of ESRD were important predictors, only recipient age was identified as a statistically significant predictor.

**Table 5.** Relative Hazard of All-Cause Graft Loss by Pre-transplant and Post-transplant Characteristics.

Characteristics	Hazard ratio	95% confidence interval
Recipient age	1.021	0.985-1.059
Recipient sex (male)	1.019	0.504-2.058
Cold ischemia time	1.003	0.961-1.047
Recipient’s cause of end-stage renal disease (diabetic nephropathy)	1.476	0.783-2.782
Donor terminal eGFR	1.006	0.989-1.023
Delayed graft function	1.533	0.794-2.961
Longitudinal recipient eGFR	0.947	0.923-0.971
C-index	0.71 (SE = 0.047)	

*Note.* In total, 116 donors were 60 years of age or older. eGFR = estimated glomerular filtration rate by the Chronic Kidney Disease Epidemiology Collaboration (CKD-EPI) equation.

When assessing the association between pre- and post-transplant characteristics with DCGF using univariate models, only DGF was statistically significantly associated with DCGF (HR: 4.24; 95% CI: 1.92-9.38). Thus, our final model for DCGF included recipient age, sex, and DGF as covariates (C-index: 0.687; SE = 0.058).

**Figure 3.**
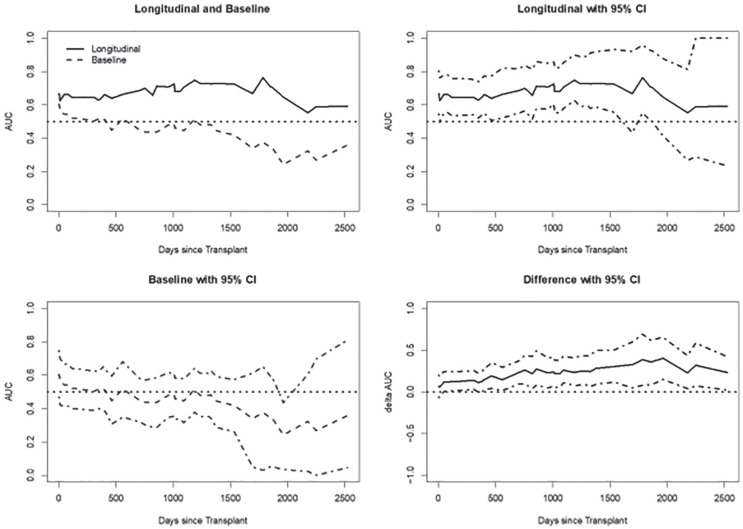
Time-dependent area under the curve for longitudinal estimated glomerular filtration rate post-transplant. *Note.* Weighted Mean Rank (WMR) curves characterizing the predictive accuracies of baseline and longitudinal eGFR over time. The first (left, top) plot shows that the accuracy of the longitudinal measurement is higher than the baseline, which is not useful for prediction (close to the null AUC value of 0.5 for a large portion). The second (right, top) plot shows the predictive accuracy of longitudinal eGFR along with the 95% bootstrap confidence interval. The third (left, bottom) plot shows the predictive accuracy of baseline eGFR along with the 95% bootstrap confidence interval. The fourth (right, bottom) plot shows the incremental accuracy of the longitudinal eGFR over the baseline eGFR along with the 95% bootstrap confidence interval. CI = confidence interval; AUC = area under the curve; eGFR = estimated glomerular filtration rate.

In our exploratory analysis, we noted that the longitudinally updated eGFR was highly predictive of outcome. The Weighted Mean Rank (WMR) estimate of the longitudinal eGFR sustained a high predictive value (WMR above 0.65 for most of the study duration), whereas the WMR estimate of baseline eGFR was not useful (WMR close to 0.5 for most of the study duration). The WMR curve based on the longitudinal eGFR and baseline eGFR as well as the difference in their prediction accuracy (and 95% bootstrap CI) are displayed in Figure 3. The bottom-right subfigure demonstrates a significantly higher prediction accuracy of the longitudinal eGFR as compared with baseline eGFR. The associated C*-index (95% bootstrap CI) for the longitudinal eGFR is 0.659 (0.540-0.764) demonstrating a good overall prediction accuracy for the entire study duration. In addition, a model described in Table 5 has a C*-index of 0.698 (95% bootstrap CI: 0.584-0.796). This analysis demonstrates that longitudinal eGFR measurements are more accurate in predicting long-term transplant outcome than a single measurement.

Because Transplant Quebec policy dictated that all donors who were ≥60 years old underwent procurement biopsies during the study period, we conducted a sensitivity analysis to verify the validity of our observations in this subgroup. An analysis restricted to this subgroup did not differ meaningfully from the observations in the entire ECD cohort (Table 6).

**Table 6.** Sensitivity Analyses, Relative Hazard of All-Cause Graft Loss in Subgroup of Kidney Transplants Recipients Whose Donors Were 60 Years of Age or Older.

Characteristics	Relative hazard of all-cause graft loss by pre-transplant characteristics		Relative hazard of all-cause graft loss by pre- and post-transplant characteristics	
	Hazard ratio	95% CI	Hazard ratio	95% CI
Recipient age	1.046	1.006-1.088	1.030	0.991-1.071
Recipient sex (male)	0.765	0.409-1.433	1.214	0.574-2.566
Cold ischemia time	1.006	0.962-1.052	1.009	0.967-1.053
Recipient’s cause of end-stage renal disease (diabetic nephropathy)	1.656	0.885-3.097	1.549	0.813-2.950
Donor terminal eGFR	1.008	0.987-1.029	1.006	0.988-1.023
Delayed graft function			1.409	0.715-2.776
Longitudinal recipient eGFR			0.950	0.926-0.974
C-index	0.596 (SE = 0.047)		0.712 (SE = 0.048)	

*Note.* In total, 116 donors were 60 years of age or older. CI = confidence interval; eGFR = estimated glomerular filtration rate by the Chronic Kidney Disease Epidemiology Collaboration (CKD-EPI) equation.

## Discussion

This study sought to address one of the greatest challenges in decision-making when offered ECD transplants—how to predict which combination of donor, recipient, and transplant characteristics will ensure the best possible kidney transplant outcome? To this end, we fit Cox regression models with *pre-transplant* donor, recipient, and transplant characteristics. Among the pre-transplant characteristics, in most multivariate analyses, we found that histopathologic features on ECD procurement biopsies were poor predictors of both short- and long-term graft outcomes. For long-term outcomes, inclusion of donor eGFR, cold ischemia time, recipient age, sex, and diabetic nephropathy as causes of ESRD marginally improved model discrimination. A model including the *post-transplant* characteristics DGF and longitudinal eGFR, in addition to *pre-transplant* characteristics, increased the prediction accuracy of overall graft survival from 0.590 (*pre-transplant* model) to 0.711 (*post-transplant* model). Moreover, this dynamic model demonstrated a high degree of prediction accuracy over time.

Prediction models for all-cause graft loss of ECD transplant recipients were developed in a conventional step-by-step fashion. Of the pre-transplant recipient characteristics, age was the most important predictor of all-cause graft loss. This finding aligns with a recent SRTR (Scientific Registry of Transplant Recipients) analysis that demonstrated how the impact of donor quality on long-term outcomes might vary by candidate condition, measured by the Estimated Post-Transplant Survival (EPTS) score. The EPTS relies on data acquired at wait-listing and considers time on dialysis, diagnosis of diabetes, prior solid organ transplant, and age.^13^

Our finding that ECD kidney quality assessment on FSWB has limited utility as a predictor of long-term outcomes is consistent with a growing body of evidence.^14^ This observation may be explained by high interobserver variability when pathologists score chronic lesions, dependence on biopsies being examined by an experienced renal pathologist or not, overrepresentation of lesions such as GS and CI in the outer cortex of the kidney,^10,15-18^ and tendency toward underrepresentation of vascular pathology including arteriosclerosis or AH (usually more prominent in arcuate and large-caliber arteries often missing in wedge biopsies).^9^ Interestingly, markedly higher discard rates for biopsied kidneys than nonbiopsied kidneys were observed in the United States in 2016, with nearly one-third of biopsied kidneys discarded, despite declining KDRI of biopsied kidneys over the past decade (from 1.61 in 2005 to 1.45 in 2016).^19^ Given the limitations and poor predictive value of donor kidney histopathology, it is hard to justify using information from procurement biopsies to decide between organ utilization and discard.

Our finding that donor terminal eGFR is not predictive of long-term transplant outcomes aligns with Young et al,^20^ who found the ECD classification useful for risk stratification of donor kidneys outside of the United States. The same study, however, reported that the predictive value of the ECD system did not exceed that provided by donor age. Moreover, terminal MDRD (Modification of Diet in Renal Disease Modification of Diet in Renal Disease) eGFR did not appear to drive the association between ECD status and graft loss.

In our cohort of ECD KTR, we found that donor characteristics associated with the short-term outcomes DGF and 1-year eGFR included donor history of diabetes and DCD status (as already included in KDRI). A sensitivity analysis using a LASSO model found only KDRI to be a statistically significant predictor of 1-year eGFR. The comprehensive KDRI score based on 10 donor characteristics was not predictive of long-term transplant outcomes when considered in Cox regression models. The U.S.-developed KDRI was studied in several Canadian populations, including Ontario, BC, and Alberta.^21-23^ While generalizable, its predictive role above and beyond a smaller number of variables, and specifically, donor age, is not convincing. Inability to observe an association between KDRI and long-term graft outcomes in our study may be related to insufficient power. In addition, our study focused on ECD KTR, resulting in a higher median KDRI in comparison with the originally used U.S. donor pool.^6^ Our analysis suggests that beyond the ECD status, which is already deemed higher risk, KDRI may not provide any additional information. It is also important to note the KDRI was reported to have insufficient prediction accuracy of long-term graft outcomes even in the SRTR,^7^ from which it had been originally derived. Given accuracy of prediction models tends to be overestimated in the population they are derived from, it is not surprising that in our patient population, KDRI was a less important predictor. Moreover, a growing body of literature finds KDRI and its derivative, Kidney Donor Profile Index (assigning a percentile score of 0-100 based on kidney donors recovered in previous years), may have resulted in overlabeling of organs as high risk leading to excessive organ discard,^7^ suggesting that caution must be exercised when considering organ discard based on clinical ECD characteristics.

Of the baseline transplant characteristics considered as predictors of all-cause graft loss, surprisingly, our analysis did not find any evidence of impact of cold ischemia time. This differs from a recent study of ECD transplants in France.^14^ The discrepancy between studies may be explained by the small percentage of DCD-ECD donors, the relatively short cold ischemia time, and the protective effect of pulsatile machine perfusion, which was used for most of the transplants in the Quebec cohort only.

A key finding of our study is that dynamic post-transplant eGFR is the most important predictor of all-cause graft loss. Analysis of eGFR decline has been shown to predict development of ESRD^24-26^ and eGFR slopes were previously shown to indicate graft failure secondary to immune-mediated injuries.^27,28^ Although post-transplant longitudinal eGFR measurements are not included in most prediction models evaluating graft outcomes, a “dynamic” approach to risk prediction for progression to kidney failure has been proposed in the context of chronic kidney disease.^29^ It is rational to assume that recipient characteristics and events influencing eGFR over the post-transplant course can similarly result in irreversible damage and affect long-term graft outcomes. Because this approach more closely simulates transplant nephrologists’ clinical monitoring practices, we believe that integration of our prediction model into electronic health records can facilitate reevaluation of ECD KTRs’ risk of graft loss on an ongoing basis. This information can facilitate timely interventions to mitigate risk of premature graft loss, and when this is not possible, to allow patient-provider communication for appropriate ESRD management planning.

We report on predictors of transplant outcomes in one of the largest single-center ECD KTR cohorts studied to date. In contrast to prior studies using preimplantation biopsies to judge donor kidney quality, our study is less vulnerable to selection bias.^9,30^ This is because, during the study period, Quebec Transplant policy mandated that procurement biopsies be conducted in all donors ≥60 years and decisions on organ utilization did not rely primarily on biopsy findings. Some limitations must also be noted. First, our effort to capture all pertinent pre-transplant determinants of donor quality in ECD KTR, including histology on FSWB, resulted in a modest sample size that was not amenable to subgroup analyses. Second, Quebec allocation practices of ECD kidneys resulted in the analytical cohort being composed of relatively older KTR with an associated higher comorbidity burden. We expect this practice to be consistent with that of other jurisdictions. Our analyses account for relevant measurable donor, recipient, and transplant characteristics. Yet, the observational nature of the study makes it vulnerable to residual confounding. For example, post-transplant donor-specific antibody (DSA) screening was implemented at our center toward the end of the study period. Consequently, while the practice in Quebec was to pursue transplantation in the absence of preformed DSA (complement-dependent cytotoxicity and/or flow cross-match negative transplants), de novo DSAs were not included among the dynamic predictors of long-term transplant outcomes.

It is likely that more ECD donors will become available given our aging population. In our study, of the pre-transplant characteristics, recipient age was most predictive of long-term transplant outcomes and longitudinal post-transplant eGFR improved model accuracy. While our models require validation in external cohorts, these findings suggest that caution should be exercised when considering organ utilization or discard based on clinical and histopathologic ECD donor characteristics alone.

## Methods

### Study Design, Population, and Data Sources

We conducted a retrospective cohort study in consecutive first-time KTR who underwent ECD transplants at MUHC between January 1, 2008 and December 31, 2014. The KTRs for whom donor procurement biopsies were not conducted or for whom FSWB were not available; who underwent multiorgan transplants, or retransplantation; who had PNF; or were missing key predictor variables, were excluded from the study. Importantly, during the study period, the policy of the Quebec organ procurement organization, Transplant Quebec, was to conduct procurement biopsies in all ECD donors older than 60 years.

Data were obtained from the MUHC Transplant Database and Transplant Quebec donor charts. Ethics approval was obtained from the MUHC research ethics board (15-192-MUHC), which granted permission to access retrospective patient and donor information. The manuscript was written in adherence with the STROBE guidelines.

### Study Endpoints

The primary long-term endpoint was time to all-cause graft loss (defined as the time from transplant to the first of any of the following: graft failure requiring return to dialysis or retransplantation, and death with graft function). Death-censored graft failure, defined as return to dialysis or retransplantation, was also studied. Patients were administratively censored at the end of study follow-up (May 1, 2015).

Short-term endpoints included DGF, defined as the need for dialysis within the first week post-transplant, and eGFR calculated by the CKD-EPI formula using creatinine measurements at 12-months (±6 weeks) post-transplant.

### Explanatory Variables

We defined “baseline” variables as any donor, recipient, and/or transplant characteristics available at the time of transplantation. These characteristics included donor demographics (age, height, weight, sex, race [white vs nonwhite]), donor comorbidities (hypertension, history of smoking, diabetes, hepatitis C infection), DCD, pre-procurement estimated glomerular filtration rate (eGFR, calculated using the CKD-EPI formula), the donor-only KDRI, and histologic features on FSWB, including GS, AH, CI, CT, and CV. On-call pathologists in the procurement centers graded these features from 0 to 3 based on increasing severity. Summary histologic scores such as Karpinski and Remuzzi scores (categorized as mild [0-3], moderate [4-6], and severe [7-12]) were also considered. Baseline recipient characteristics included demographics (age, height, weight, sex, race [white vs nonwhite]), time on dialysis (in days), and cause of ESRD. Baseline transplant characteristics included transplant era (≤2011, >2011), cold ischemia time, pulsatile machine perfusion, type of induction therapy (categorized as non-lymphocyte-depleting agent, lymphocyte-depleting agent, or none), maintenance immunosuppression regimen (categorized as triple agent (tacrolimus, mycophenolate, prednisone), dual agent (tacrolimus, mycophenolate) or other, and human leukocyte antigen (HLA) mismatch (1-2, 3-4, 5-6). Finally, among the post-transplant characteristics, we considered DGF, defined as the need for dialysis during the first week after transplant, and recipient post-transplant longitudinal eGFR.

### Statistical Analysis

We performed descriptive analyses on the baseline variables (frequencies and proportion for categorical variables and mean/standard deviation for continuous variables) and Kaplan-Meier survival curve for time to all-cause graft loss. To estimate the role of pre-transplant donor characteristics in determining post-transplant outcomes, we fit several prediction models.

### Short-Term Outcome Models

#### Pre-transplant prediction model

When considering studying short-term outcomes, we found that only 3 participants experienced PNF, which was deemed too small to study this endpoint and were excluded from the analysis. We conducted logistic regression to estimate risk for DGF and linear regression to predict *one-year post-transplant* eGFR.

### Long-Term Outcome Models

#### Pre-transplant survival model

We fit a Cox proportional hazards model to characterize all-cause graft loss and DCGF. Very few donors or recipients had missing values for important covariates (see Table 1). Therefore, missing categorical variables were replaced by the most frequent or baseline (lowest risk) category. The missing continuous variables were replaced by the mean of the available observations.

Our objective was to provide a prediction model that would accurately predict time to all-cause graft loss or DCGF, be easy to interpret, and be useful to support decisions on organ utilization pre-transplant. Considering transplant outcomes are also dependent on recipient characteristics, we decided a priori to include recipient age and sex in the multivariable model. Other donor and transplant variables that were included in the prediction model regardless of their strength of association in univariate analysis were donor pre-procurement eGFR and cold ischemia time.^14,31-33^ To identify additional predictors, we first assessed univariate associations via Cox proportional hazards models between the outcome and several donor (biopsy lesions [GS, AH, CI, CT, CV], Remuzzi and Karpinski scores, KDRI, and proteinuria), recipient (cause of ESRD), and transplant characteristics (pulsatile machine perfusion, induction therapy, maintenance immune suppression therapy, and HLA mismatch). The variables that had a strong association with the outcome (as indicated by a *P* value of ≤ .2) were retained for further exploration in a multivariate Cox proportional hazards model. To maintain an expected number of events per variable between 5 and 10,^11^ we restricted the number of variables in the multivariate prediction model to be between 5 and 10, including levels of categorical variables. The validity of proportional hazards assumption was assessed using scaled Schoenfeld residuals. Overall prediction accuracy of the models was assessed using Harrell’s C-index and a novel accuracy measure C*-index.^34^ C*-index is a recently proposed accuracy index, particularly suitable for longitudinal covariates as predictors of a survival outcome.

#### Post-transplant survival model with longitudinal eGFR measurements

Because long-term ECD transplant outcomes may be influenced by post-transplant factors, we also wanted to provide a prediction model that would be able to dynamically capture changes in the risk of graft failure post-transplant. Recently, dynamic changes in eGFR have been incorporated in prediction models informing risk of ESRD.^29^ In this context, we were interested to characterize the incremental value of post-transplant longitudinal eGFR measurements for predicting time to all-cause graft loss. The longitudinal eGFR was based on serum creatinine measured repeatedly starting from the first post-transplant day. To impute missing creatinine measurements and to standardize the measurement to a 3-month measurement window, we carried the last observation forward.

We included the longitudinal eGFR in a Cox proportional hazards model that already included baseline characteristics to formally assess the incremental accuracy of post-transplant eGFR in predicting time to all-cause graft loss. We used Harrell’s C-index and C*-index to assess the overall prediction accuracy of the model. As an added visual tool, we used the recently proposed WMR estimator^35^ to show the prediction accuracy of a model that includes longitudinal eGFR measurements. The nonparametric WMR curve is a generalization of the conventional C-index and can characterize the accuracy in a longitudinal fashion to reveal potentially important time-dependent variation in prediction accuracy.

Finally, given Quebec Transplant policy to perform biopsies in all donors who were ≥60 years old, we performed a sensitivity analysis to verify the validity of our findings in this subgroup. For all models, *P* value of <.05 was considered statistically significant.

## Supplemental Material

SUPLLEMENTARY_INFORMATION_1 – Supplemental material for Predicting Clinical Outcome in Expanded Criteria Donor Kidney Transplantation: A Retrospective Cohort Study Click here for additional data file. Supplemental material, SUPLLEMENTARY_INFORMATION_1 for Predicting Clinical Outcome in Expanded Criteria Donor Kidney Transplantation: A Retrospective Cohort Study by Paramita Saha-Chaudhuri, Carly Rabin, Jean Tchervenkov, Dana Baran, Justin Morein and Ruth Sapir-Pichhadze in Canadian Journal of Kidney Health and Disease
