# Supplementary material for: Predicting Clinical Outcome in Expanded Criteria Donor Kidney Transplantation: A Retrospective Cohort Study
Source: Can J Kidney Health Dis. 2020 Jun 24;7:2054358120924305. doi: 10.1177/2054358120924305 (PMC7315672; doi:10.1177/2054358120924305)
Supplement: SUPLLEMENTARY_INFORMATION_1 – Supplemental material for Predicting Clinical Outcome in Expanded Criteria Donor Kidney Transplantation: A Retrospective Cohort Study [file SUPLLEMENTARY_INFORMATION_1.pdf]

**SUPLLEMENTARY INFORMATION 1: Descriptive Statistics for 1-Year Post-Transplant Estimated Glomerular Filtration Rate**

| Summary | Min | Q1 | Median | Mean | Q3 | Max | SD |
|---|---|---|---|---|---|---|---|
| Value | 11.02 | 34.26 | 42.77 | 45.29 | 55.37 | 104.30 | 17.76 |

**Distribution of 1-year post-transplant eGFR**

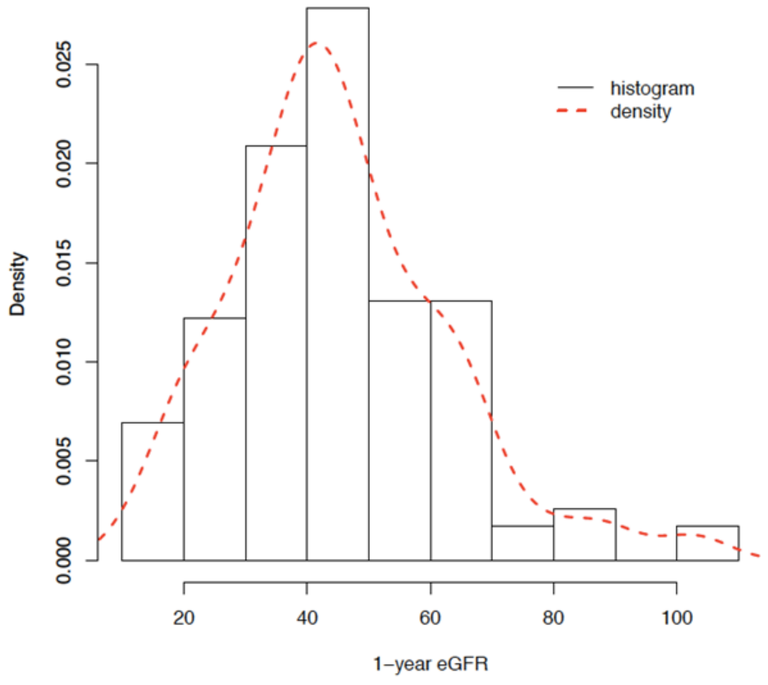


Abbreviations: Min, minimum; Q1, first quartile; Q3, third quartile; Max, maximum; SD, standard deviation
